# Demographic and socioeconomic factors associated with multiple morbidities at 40 years of age: the 1982 Pelotas (Brazil) birth cohort

**DOI:** 10.1590/0102-311XEN024625

**Published:** 2025-11-10

**Authors:** Luísa Silveira da Silva, Débora Vergara Ferro, Glaucia Treichel Heller, Ana Paula Oliveira Rosses, Priscila Moreira Vargas, Fernando C. Wehrmeister, Bernardo Lessa Horta

**Affiliations:** 1 Universidade Federal de Pelotas, Pelotas, Brasil.

**Keywords:** Morbidity, Noncommunicable Diseases, Socioeconomic Factors, Morbidade, Doenças Não Transmissíveis, Fatores Socioeconômicos, Morbilidad, Enfermedades No Transmisibles, Factores Socioeconómicos

## Abstract

This cross-sectional study assessed the association of demographic and socioeconomic variables with comorbidities at 40 years of age in participants of the 1982 Pelotas (Brazil) birth cohort. At age 40, study participants were invited to visit the research clinic to be examined and answered an online questionnaire. Subjects reported medical diagnosis of several morbidities that were grouped according to the 11th revision of the International Classification of Diseases into four groups (endocrine, cardiovascular, musculoskeletal, and allergic/respiratory). Ordinal logistic regression was used to estimate the proportional odds ratio, and Brant test to check the proportional odds assumption. Latent class analysis was used to identify multimorbidity patterns, and their association with demographic and socioeconomic factors was evaluated via multinomial logistic regression. A total of 2,986 participants were included in this study. At least one endocrine disorder was reported by 48.1% participant, cardiovascular morbidities by 26.6%, allergic/respiratory morbidities by 59%, and musculoskeletal morbidities by 32.5% of participants. In the latent class analysis, three morbidity patterns were identified: relatively healthy, metabolic and allergic/respiratory. The odds of being in a higher category of number of cardiovascular morbidities was higher among blacks (OR = 1.79; 95%CI: 1.43; 2.24). Notably, lower socioeconomic status was associated with a lower odds of being in a higher category of number of allergic/respiratory morbidities (OR = 0.59; 95%CI: 0.47; 0.74) and a higher odds of being in a higher category of number of cardiovascular and musculoskeletal morbidities. Our findings suggest that multiple morbidities occur in different directions depending on the socioeconomic and educational levels.

## Abstract (pt)

Este estudo transversal avaliou associações de variáveis demográficas e socioeconômicas com o acúmulo de morbidades aos 40 anos de idade em participantes da coorte de nascimentos de Pelotas (Brasil) de 1982. Aos 40 anos de idade, os participantes do estudo foram convidados a visitar a clínica de pesquisa para serem examinados e responderam a um questionário online. Os sujeitos relataram diagnósticos médicos de diversas morbidades, que foram agrupadas de acordo com a 11ª revisão da Classificação Internacional de Doenças em quatro grupos (endócrino, cardiovascular, musculoesquelético e alérgico/respiratório). Utilizou-se a regressão logística ordinal para estimar a razão de chances proporcional e aplicou-se o teste de Brant para verificar a suposição de chances proporcionais. Utilizou-se a análise de classe latente para identificar padrões de multimorbidade e avaliou-se associações com fatores demográficos e socioeconômicos por meio de regressão logística multinomial. Ao total, 2.986 participantes foram incluídos no presente estudo. Um total de 48,1% relataram pelo menos um distúrbio endócrino, 26,6% relataram morbidades cardiovasculares, 59% morbidades alérgicas/respiratórias e 32,5% morbidades musculoesqueléticas. Na análise de classe latente, três padrões de morbidade foram identificados: relativamente saudável, metabólico e alérgico/respiratório. A chance de pertencer a uma categoria mais alta de morbidades cardiovasculares foi maior entre os negros (OR = 1,79; IC95%: 1,43; 2,24), enquanto o nível socioeconômico mais baixo se associou à menor chance de pertencer a uma categoria mais alta de morbidades alérgicas/respiratórias (OR = 0,59; IC95%: 0,47; 0,74) e a maior chance de pertencer a uma categoria mais alta de morbidades cardiovasculares e musculoesqueléticas. Nossos achados sugerem que múltiplas morbidades ocorrem em diferentes direções, dependendo dos níveis socioeconômicos e educacionais.

## Abstract (es)

Este estudio transversal evaluó la asociación de variables demográficas y socioeconómicas con la acumulación de morbilidades a los 40 años de edad en participantes de la cohorte de nacimiento de Pelotas (Brasil) de 1982. A los 40 años, se invitó a los participantes del estudio a visitar la clínica de investigación para ser examinados y completar un cuestionario en línea. Los sujetos reportaron diagnósticos médicos de diversas morbilidades, que fueron agrupadas según la 11ª revisión de la Clasificación Internacional de Enfermedades en cuatro categorías (endocrinas, cardiovasculares, musculoesqueléticas y alérgicas/respiratorias). Se utilizó la regresión logística ordinal para estimar la *odds ratio* proporcional, y la prueba de Brant para verificar la hipótesis de probabilidad proporcional. Se utilizó el análisis de clases latentes para identificar patrones de multimorbilidad y se evaluó su asociación con factores demográficos y socioeconómicos mediante regresión logística multinomial. En el presente estudio se incluyeron 2.986 participantes. El 48,1% informó de al menos un trastorno endocrino, el 26,6% de morbilidades cardiovasculares, el 59% de morbilidades alérgicas/respiratorias y el 32,5% de morbilidades musculoesqueléticas. En el análisis de clases latentes, se identificaron tres patrones de morbilidad: relativamente saludable, metabólico y alérgico/respiratorio. La probabilidad de encontrarse en una categoría más alta de número de morbilidades cardiovasculares era mayor entre las personas de raza negra (OR = 1,79; IC95%: 1,43; 2,24). Mientras que un menor nivel socioeconómico se asoció con menores probabilidades de pertenecer a una categoría superior en el número de morbilidades alérgicas/respiratorias (OR = 0,59; IC95%: 0,47; 0,74) y mayores probabilidades de pertenecer a una categoría superior en el número de morbilidades cardiovasculares y musculoesqueléticas. Nuestros hallazgos sugieren que las multimorbilidades ocurren en direcciones diferentes según los niveles socioeconómicos y educativos.

## Introduction

Cardiovascular diseases, cancer, diabetes and chronic respiratory diseases are the leading cause of death, being responsible for 74% of deaths worldwide ^1^. About 15 million of these deaths are considered premature, occurring in individuals aged 30 to 70 years, and more than 85% of the premature deaths by noncommunicable diseases (NCDs) occur in low- and middle-income countries ^2^.

Subjects living in socioeconomic disadvantage are more likely to develop NCDs, mainly because of the lower access to information and resources to maintain good health ^3^. A systematic review found that people in the lowest income level had 4.4 times higher odds of multimorbidity than those in the highest level ^4^. A systematic review about the social determinants of multimorbidity showed that cardiometabolic, musculoskeletal, mental and respiratory morbidities were the most prevalent, especially among poor individuals ^5^. On the other hand, higher rates of respiratory diseases were related to middle and high socioeconomic status ^5^.

Evaluating morbidities in early middle age is crucial due to early prevention. While regular monitoring of risk factors and consideration of long-term risk are emphasized for adults aged 20 to 39 years, for those aged 40 and over interventions should consider that many people already have multiple morbidities ^6^.

Although several studies have evaluated the association of socioeconomic status with morbidities in adulthood, most of them were carried out in high-income countries ^4^ ^,^ ^5^ ^,^ ^7^. It is relevant to evaluate this association in low and middle-income countries considering the existent differences regarding socioeconomic structure, access to health care services and health determinants; all of which may have their effects modified by the country income ^2^. In low- and middle-income countries, socioeconomic status is negatively associated with tobacco and alcohol consumption and positively with physical inactivity and ultra-processed food intake, risk factors commonly associated with the NCDs development. Notably, these associations between socioeconomic status and risk factors follow an opposite direction in high-income countries ^8^ ^,^ ^9^. A multicenter study (*Brazilian Longitudinal Study of Adult Health* - ELSA-Brasil, acronym in Portuguese) in Brazil found an overall prevalence of multimorbidity of 70% among adults in 2010 (median age: 51 years) ^10^. On the other hand, evidence from the *Brazilian National Health Survey* (PNS, acronym in Portuguese) showed that the prevalence of multimorbidity among adults from 30 to 59 years increased from 24.4% in 2013, to 27.7% in 2019 ^11^.

This study aimed to evaluate the association of demographic and socioeconomic factors with the number of multiple morbidities at 40 years of age in participants of the 1982 Pelotas (Brazil) birth cohort.

## Methods

In 1982, maternity hospitals in Pelotas (a city in southern Brazil) were daily visited. All births were identified and live births whose family lived in the urban area of Pelotas were examined and their mothers interviewed soon after childbirth (n = 5,914). These subjects have been prospectively followed at different ages ^12^. From August 2022 to June 2023, we tried to follow up all cohort members, who were searched using multiple strategies, totaling 3,087 participants, which added to the 395 identified deaths, represented a follow-up rate of 58.9%. An online questionnaire was sent to all participants who were also invited to attend the research clinic to be examined and collect biological samples. This study included participants with information at 40 years on morbidities, socioeconomic and demographic variables, weight and height measurements (n = 2,986). Pregnant women or those with suspected pregnancy were excluded for weight and height measurements, as well as those with some physical limitation or cognitive impairment that would prevent data collection of any information of interest.

Participants were asked about medical diagnosis (based on lifetime history) of the following morbidities: ischemic disease (stroke, angina, or heart attack); arrhythmia or valvular heart disease; hypertension; thyroid pathologies (hypothyroidism, hyperthyroidism, or Hashimoto’s thyroiditis); diabetes; allergic rhinitis; skin allergy or eczema; asthma (based on the report of wheezing ^13^); rheumatic diseases (including arthritis and arthrosis), and chronic pain (neck pain, back pain, or low back pain). Body weight was measured using a scale attached to the Bod Pod (COSMED: the Metabolic Company, https://www.cosmed.com/en/) and height was measured with a portable stadiometer (Harpenden; https://holtain.co.uk/). Obesity was defined as a body mass index (BMI) ≥ 30kg/m².

Morbidities were subsequently grouped according to the 11th revision of the International Classification of Diseases (ICD-11) into the following groups: endocrine (thyroid pathologies, diabetes, and obesity); cardiovascular (ischemic disease, arrhythmia, valvular heart disease, and hypertension); musculoskeletal (arthritis, arthrosis and chronic pain), and allergic and respiratory (allergic rhinitis, skin allergy, or eczema and asthma). To calculate the general prevalence of each group, variables were created by combining the prevalence of diseases in each group, if a given participant had at least one of the diseases in the group, they were considered a prevalent case for the given group. We also evaluated the number of morbidities in each group, these variables were categorized into zero, one, two and three or more morbidities.

Demographic and socioeconomic variables evaluated included sex, self-reported skin color using the categories used by Brazilian Institute of Geography and Statistics (IBGE, acronym in Portuguese), participants who identified as yellow or Indigenous were excluded due to the small sample (n = 10), schooling in complete years, and socioeconomic status evaluated using the economic classification criteria of the Brazilian Association of Research Companies (ABEP, acronym in Portuguese) ^14^.

A theoretical model was used to choose the confounding variables. Sex and skin color were considered as distal determinants of the outcomes. Education and income were evaluated as more proximal determinants. Since behavioral factors were considered as mediators in the relationship between demographic and socioeconomic and morbidities, these factors were not included in the adjusted model.

Descriptive analyses are shown by absolute and relative frequencies and their respective 95% confidence interval (95%CI). Ordinal logistic regression was used to assess the association of demographic and socioeconomic variables with the number of multimorbidity in each group, and the proportional odds assumption was evaluated using the Brant test. Crude and adjusted analysis were conducted adjusting for confounders based on the theoretical model; sex and skin color were included. Education and income were not included in the same model due to the collinearity among these variables. Such collinearity was assessed using the variance inflation factor (VIF) and showed a moderate collinearity (VIF = 4.9).

Latent class analysis was used to identify morbidities patterns, the number of classes was defined based on the relative fit of the models, according to Akaike information criteria (AIC) and Bayesian information criterion (BIC). The probability of each morbidity occurring within different latent classes was estimated. Then, each participant was assigned to a class if their previous probability of having a morbidity was greater than 0.5, which enabled to interpret the classes as specific morbidities patterns. Thus, a categorical variable was generated to represent the three identified patterns. This outcome was evaluated according to the demographic and socioeconomic variables via both crude and adjusted multinomial logistic regression, applying the same adjustment model previously mentioned in the ordinal logistic regression analysis.

Inverse probability weighting was applied to estimate selection bias using perinatal variables (sex, maternal skin color, maternal education and family income). The absolute standardized differences before and after weighting using weights calculated from the propensity score to be a loss were estimated and the associations results were reassessed to check if the selection bias affected these results (Tables 1 and 2). All analyses were conducted using the Stata 15.0 program (https://www.stata.com).

The Research Ethics Committee of the Faculty of Medicine of the Federal University of Pelotas (UFPel) approved this study, under protocol number 58079722.8.0000.5317. All participants signed an informed consent form.

**Table 1:** Absolute standardized differences between losses and non-losses before and after weighting using weights calculated from the propensity score to be a loss.

Variable	Before weighting			After weighting		
	Mean in followed	Mean in not followed	Standardized difference	Mean in followed	Mean in not followed	Standardized difference
Sex	1.42	1.54	0.244	1.49	1.49	0.000
Maternal skin color	1.18	1.18	0.015	1.18	1.18	0.001
Maternal education	6.18	6.78	0.143	6.48	6.49	0.002
(Maternal education)²	54.62	64.41	0.135	59.42	59.57	0.002
Family income	2.20	2.33	0.127	2.26	2.27	0.001
(Family income)²	5.93	6.53	0.100	6.22	6.23	0.001
(Sex) × (maternal skin color)	1.68	1.81	0.159	1.75	1.75	0.005
(Sex) × (maternal education)	8.81	10.56	0.237	9.65	9.74	0.012
(Sex) × (family income)	3.13	3.62	0.246	3.36	3.39	0.013
(Maternal skin color) × (maternal education)	6.99	7.69	0.146	7.31	7.38	0.014
(Maternal skin color) × (family income)	2.50	2.65	0.123	2.57	2.58	0.005
(Maternal education) × (family income)	16.26	18.67	0.129	17.42	17.48	0.003

**Table 2:** Results after inverse probability weighting: association between multiple morbidities and demographic and socioeconomic factors in individuals at 40 years of age. 1982 Pelotas (Brazil) birth cohort, 2022/2023.

Variable	Multiple morbidities OR (95%CI)							
	Endocrine		Cardiovascular		Musculoskeletal		Allergic/Respiratory	
	Crude	Adjusted	Crude	Adjusted	Crude	Adjusted	Crude	Adjusted
Sex								
Male	1.00 Reference	1.00 Reference	1.00 Reference	1.00 Reference	1.00 Reference	1.00 Reference	1.00 Reference	1.00 Reference
Female	1.13 (0.95; 1.36)	1.13 (0.95; 1.36)	1.07 (0.89; 1.27)	1.08 (0.91; 1.29)	0.93 (0.79; 1.08)	0.92 (0.79; 1.08)	1.10 (0.96; 1.26)	1.10 (0.96; 1.26)
Skin color								
White	1.00 Reference	1.00 Reference	1.00 Reference	1.00 Reference	1.00 Reference	1.00 Reference	1.00 Reference	1.00 Reference
Black	1.20 (0.95; 1.52)	1.20 (0.95; 1.53)	1.75 (1.39; 2.19)	1.76 (1.40; 2.20)	0.82 (0.66; 1.02)	0.82 (0.66; 1.02)	0.75 (0.62; 0.90)	0.75 (0.63; 0.91)
Mixed-race	1.25 (0.93; 1.68)	1.25 (0.93; 1.68)	1.18 (0.89; 1.58)	1.18 (0.89; 1.57)	0.96 (0.74; 1.24)	0.96 (0.74; 1.24)	1.10 (0.89; 1.36)	1.10 (0.89; 1.36)
Socioeconomic level (ABEP)								
AB	1.00 Reference	1.00 Reference	1.00 Reference	1.00 Reference	1.00 Reference	1.00 Reference	1.00 Reference	1.00 Reference
C	1.20 (0.99; 1.46)	1.20 (0.98; 1.46)	1.28 (1.06; 1.54)	1.24 (1.02; 1.50)	1.11 (0.94; 1.31)	1.13 (0.95; 1.34)	0.81 (0.70; 0.94)	0.82 (0.71; 0.95)
DE	1.25 (0.94; 1.66)	1.23 (0.92; 1.65)	1.50 (1.12; 1.99)	1.36 (1.02; 1.83)	1.49 (1.16; 1.92)	1.56 (1.21; 2.02)	0.58 (0.47; 0.73)	0.60 (0.48; 0.76)
Education (years of study)								
≥ 12	1.00 Reference	1.00 Reference	1.00 Reference	1.00 Reference	1.00 Reference	1.00 Reference	1.00 Reference	1.00 Reference
9-11	1.13 (0.93; 1.38)	1.13 (0.93; 1.38)	1.40 (1.15; 1.70)	1.34 (1.10; 1.63)	1.20 (1.00; 1.43)	1.23 (1.03; 1.47)	0.78 (0.67; 0.91)	0.79 (0.68; 0.93)
0-8	1.29 (0.99; 1.67)	1.26 (0.97; 1.64)	1.36 (1.06; 1.75)	1.26 (0.97; 1.63)	1.40 (1.12; 1.75)	1.47 (1.17; 1.85)	0.52 (0.43; 0.63)	0.53 (0.43; 0.64)

95%CI: 95% confidence interval; ABEP: Brazilian Association of Research Companies; OR: odds ratio.

Note: adjusted for sex and skin color.

## Results

At 40 years, 3,087 subjects were interviewed and 2,986 were included in this study. Table 3 shows that 54.6% of the participants were women and most self-reported as being white (73.6%). Regarding socioeconomic conditions, 46.9% belonged to class C and almost half of the participants had at least 12 years of schooling (50.3%). The general prevalence of each group of morbidities was 48.1% (95%CI: 46.0; 50.2) for endocrine diseases, 26.6% (95%CI: 25.0; 28.2) for cardiovascular morbidities, 59% (95%CI: 57.2; 60.7) for allergic/respiratory morbidities, and 32.5% (95%CI: 30.8; 34.2) for musculoskeletal morbidities.

**Table 3:** Description of the sample according to socioeconomic and demographic characteristics at 40 years of age. 1982 Pelotas (Brazil) birth cohort, 2022/2023.

Characteristics	n (%)	95%CI
Sex [n = 2,986]		
Male	1,357 (45.4)	43.7; 47.2
Female	1,629 (54.6)	52.7; 56.3
Skin color [n = 2,951]		
White	2,173 (73.6)	72.0; 75.2
Black	461 (15.6)	14.4; 17.0
Mixed-race	317 (10.7)	9.7; 11.9
Socioeconomic level (ABEP) [n = 2,975]		
AB	1,214 (40.8)	39.0; 42.6
C	1,395 (46.9)	45.1; 48.7
DE	366 (12.3)	11.2; 13.5
Education (years of study) [n = 2,950]		
≥ 12	1,485 (50.3)	48.5; 52.1
9-11	992 (33.6)	31.9; 35.4
0-8	473 (16.0)	14.8; 17.4
Endocrine morbidities		
Thyroid pathologies [n = 2,290]	103 (4.5)	3.7; 5.4
Diabetes [n = 2,945]	185 (6.3)	5.5; 7.2
Obesity [n = 2,594]	908 (35.0)	33.2; 36.9
Overall * [n = 2,236]	1,075 (48.1)	46.0; 50.2
Cardiovascular morbidities		
Ischemic disease [n = 2,927]	60 (2.0)	1.6; 2.6
Arrhythmia/Valvular heart disease [n = 2,816]	70 (2.5)	2.0; 3.1
Hypertension [n = 2,933]	656 (22.4)	20.9; 23.9
Overall * [n = 2,788]	741 (26.6)	25.0; 28.2
Musculoskeletal morbidities		
Arthritis/Arthrosis [n = 2,939]	136 (4.6)	3.9; 5.4
Chronic back pain [n = 2,942]	893 (30.4)	28.7; 32.0
Overall * [n = 2,930]	951 (32.5)	30.8; 34.2
Allergic/respiratory morbidities		
Allergic rhinitis [n = 2,929]	1,048 (35.8)	34.1; 37.5
Skin allergy/Eczema [n = 2,927]	611 (20.9)	19.4; 22.4
Asthma [n = 2,949]	990 (33.6)	31.9; 35.3
Overall * [n = 1,731]	1,731 (59.0)	57.2; 60.7

95%CI: 95% confidence interval; ABEP: Brazilian Association of Research Companies.

* Prevalence of at least one disease in the group.

Table 4 shows the prevalence of morbidities in each group. About 16% (95%CI: 14.9; 18.3) of the studied population had no morbidity, while 24.2% (95%CI: 22.3; 26.2) had two and 6.9% (95%CI: 5.8; 8.1) had five or more morbidities. Most participants (58.9%; 95%CI: 56.7; 61.0) did not have any endocrine disease, whereas 5.1%(95%CI: 4.2; 6.2) had two endocrine diseases. Regarding cardiovascular morbidities, most subjects did not have any morbidity (74.8%; 95%CI: 73.1; 76.4) and 23.8% (95%CI: 22.2; 25.4) had one. Concerning musculoskeletal morbidities, 29.6% (95%CI: 28.0; 31.3) reported one morbidity. For allergic/respiratory diseases, 41.5% (95%CI: 39.7; 43.3) did not have any morbidity, while 17.7% (95%CI: 16.4; 19.2) and 6.8% (95%CI: 6.0; 7.8) had two and three morbidities, respectively.

**Table 4:** Prevalence of comorbidities in each group. 1982 Pelotas (Brazil) birth cohort, 2022/2023.

Morbidity group	Number of comorbidities [% (95%CI)]					
	0	1	2	3	4	5
Endocrine *	58.9 (56.7; 61.0)	36.0 (33.9; 38.1)	5.1 (4.2; 6.2)	0.1 (0.01; 0.04)	-	-
Cardiovascular **	74.8 (73.1; 76.4)	23.8 (22.2; 25.4)	1.4 (1.0; 1.9)	-	-	-
Musculoskeletal ***	67.8 (66.0; 69.4)	29.6 (28.0; 31.3)	2.7 (2.1; 3.3)	-	-	-
Allergic/Respiratory ^#^	41.5 (39.7; 43.3)	34.0 (32.3; 35.7)	17.7 (16.4; 19.2)	6.8 (6.0; 7.8)	-	-
Overall	16.5 (14.9; 18.3)	26.7 (24.7; 28.7)	24.2 (22.3; 26.2)	17.0 (15.4; 18.8)	8.7 (7.5; 10.1)	6.9 (5.8; 8.1)

95%CI: 95% confidence interval^.^

* Thyroid pathologies, diabetes, and obesity;

** Ischemic disease, arrhythmia/valvular heart disease, and hypertension;

*** Arthritis/arthrosis, chronic back pain;

^#^ Allergic rhinitis, skin allergy/eczema and asthma.

The results of the Brant test showed that the proportional odds assumption was not violated (p > 0.05; data not shown). Table 5 shows that having 8 or less years of schooling was positively associated with the odds of being in a higher category of number of endocrine diseases. After controlling for confounding, the magnitude of the association slightly decreased and the confidence interval included the nullity (OR = 1.25; 95%CI: 0.97; 1.62). The odds of being in a higher category of number of cardiovascular morbidities was 79% (95%CI: 1.43; 2.24) higher among black subjects in relation to the white ones. Regarding cardiovascular morbidities, the odds was higher among those with socioeconomic levels C (OR = 1.25; 95%CI: 1.04; 1.51) and D/E (OR = 1.53; 95%CI: 1.16; 2.01), in relation to A/B. Regarding allergic/respiratory morbidities, black participants had a 23% (95%CI: 0.64; 0.93) lower odds of being in a higher category of number of morbidities, than white ones. Compared with levels A/B, those in the socioeconomic levels C and D/E had 18% and 41% lower odds of being in a higher category of number of morbidities. Schooling was also associated with a lower odds of being in a higher category of number of allergic/respiratory morbidities, (≤ 8 years of study - OR = 0.51; 95%CI: 0.41; 0.62; reference category: > 12 years of study), whereas for musculoskeletal morbidities the odds of being in a higher category of number of morbidities was higher among those at socioeconomic level D/E (OR = 1.56; 95%CI: 1.22; 2.01), and those with lower schooling.

**Table 5:** Association between multiple morbidities and demographic and socioeconomic factors in individuals at 40 years of age. 1982 Pelotas (Brazil) birth cohort, 2022/2023.

Variable	Multiple morbidities OR (95%CI)							
	Endocrine		Cardiovascular		Musculoskeletal		Allergic/respiratory	
	Crude	Adjusted	Crude	Adjusted	Crude	Adjusted	Crude	Adjusted
Sex								
Male	1.00 Reference	1.00 Reference	1.00 Reference	1.00 Reference	1.00 Reference	1.00 Reference	1.00 Reference	1.00 Reference
Female	1.12 (0.94; 1.34)	1.13 (0.94; 1.35)	1.10 (0.92; 1.30)	1.10 (0.92; 1.31)	0.93 (0.80; 1.09)	0.92 (0.79; 1.08)	1.10 (0.96; 1.26)	1.10 (0.96; 1.25)
Skin color								
White	1.00 Reference	1.00 Reference	1.00 Reference	1.00 Reference	1.00 Reference	1.00 Reference	1.00 Reference	1.00 Reference
Black	1.24 (0.98; 1.58)	1.24 (0.98; 1.58)	1.79 (1.43; 2.24)	1.79 (1.43; 2.25)	0.83 (0.66; 1.03)	0.83 (0.66; 1.03)	0.77 (0.64; 0.93)	0.77 (0.64; 0.93)
Mixed-race	1.24 (0.93; 1.65)	1.23 (0.93; 1.65)	1.18 (0.89; 1.57)	1.18 (0.89; 1.56)	0.97 (0.75; 1.24)	0.96 (0.75; 1.24)	1.12 (0.90; 1.38)	1.12 (0.90; 1.40)
Socioeconomic level (ABEP)								
AB	1.00 Reference	1.00 Reference	1.00 Reference	1.00 Reference	1.00 Reference	1.00 Reference	1.00 Reference	1.00 Reference
C	1.17 (0.97; 1.42)	1.16 (0.96; 1.41)	1.25 (1.04; 1.51)	1.22 (1.01; 1.48)	1.11 (0.94; 1.31)	1.13 (0.96; 1.37)	0.81 (0.70; 0.94)	0.82 (0.71; 0.95)
DE	1.27 (0.96; 1.68)	1.21 (0.90; 1.61)	1.53 (1.16; 2.01)	1.38 (1.04; 1.84)	1.48 (1.16; 1.89)	1.56 (1.22; 2.01)	0.56 (0.45; 0.70)	0.59 (0.47; 0.74)
Education (years of study)								
≥ 12	1.00 Reference	1.00 Reference	1.00 Reference	1.00 Reference	1.00 Reference	1.00 Reference	1.00 Reference	1.00 Reference
9-11	1.16 (0.96; 1.42)	1.14 (0.93; 1.39)	1.39 (1.15; 1.69)	1.34 (1.10; 1.63)	1.21 (1.02; 1.44)	1.23 (1.03; 1.47)	0.76 (0.65; 0.88)	0.77 (0.66; 0.89)
0-8	1.28 (1.00; 1.65)	1.25 (0.97; 1.62)	1.34 (1.05; 1.71)	1.24 (0.96; 1.60)	1.42 (1.14; 1.76)	1.49 (1.19; 1.86)	0.50 (0.41; 0.62)	0.51 (0.41; 0.62)

95%CI: 95% confidence interval; ABEP: Brazilian Association of Research Companies.

Note: adjusted for sex and skin color.

Table 6 shows the predicted probabilities for the occurrence of each morbidity across the three latent classes. The first pattern was labeled “relatively healthy” due to the low probabilities of occurrence of morbidities among participants. This class included most of the sample (69%), and a 36.2% of multimorbidity was observed. The second pattern showed higher probabilities to obesity (72%; 95%CI: 64.0; 78.8) and hypertension (70.1%; 95%CI: 59.2; 79.1), it was labeled “metabolic”. The third pattern was identified as “allergic/respiratory” due to the high probabilities of allergic rhinitis (80.6%; 95%CI: 67.9; 89.1) and asthma 59.8%; 95%CI: 52.2; 67.0) occurrence. The “metabolic” and “allergic/respiratory” patterns included 14.1 and 16.9% of the sample respectively, both with 100% of multimorbidity and with an average of more than three morbidities.

**Table 6:** Class-specific predicted probabilities from three latent class models of morbidities.

Morbidity	Classes		
	Relatively healthy	Metabolic	Allergic/Respiratory
	% (95%CI)	% (95%CI)	% (95%CI)
Thyroid pathologies	5.3 (4.2; 6.7)	2.3 (1.0; 5.4)	3.0 (1.4; 6.2)
Diabetes	3.0 (2.1; 4.4)	24.8 (19.8; 30.7)	-
Obesity	26.2 (23.5; 29.2)	72.0 (64.0; 78.8)	28.0 (21.7; 35.4)
Ischemic disease	0.05 (0.02; 1.5)	9.0 (6.4; 12.7)	0.07 (0.02; 3.4)
Arrhythmia/Valvular heart disease	2.7 (2.0; 3.7)	0.05 (0.01; 3.0)	2.9 (1.5; 5.5)
Hypertension	12.3 (10.0; 15.1)	70.1 (59.2; 79.1)	12.1 (7.4; 19.2)
Allergic rhinitis	20.6 (17.3; 24.3)	41.3 (34.4; 48.6)	80.6 (67.9; 89.1)
Skin allergy/Eczema	9.0 (6.8; 11.8)	27.4 (21.9; 33.7)	53.9 (44.8; 62.6)
Asthma	20.2 (17.4; 23.4)	52.7 (46.0; 59.4)	59.8 (52.2; 67.0)
Arthritis/Arthrosis	1.2 (0.06; 2.2)	15.2 (11.5; 19.8)	6.4 (3.9; 10.2)
Back pain	20.4 (17.9; 23.2)	50.7 (44.4; 57.0)	44.3 (38.0; 50.7)
Class proportion (%)	69.0	14.1	16.9
Multimorbidity (%)	36.2	100.0	100.0
Number of conditions (mean)	1.2	4.2	3.2

95%CI: 95% confidence interval.

The association between the patterns of morbidity and demographic and socioeconomic factors are described in Table 7. In relation to the “relatively healthy” pattern, black skin color was associated with higher odds of belonging to “metabolic” pattern (OR = 1.46; 95%CI: 1.12; 1.90), while for the “allergic/respiratory” pattern the association was inverse (OR = 0.62; 95%CI: 0.46; 0.85), compared to white skin color. The associations with socioeconomic level and education follow a similar pathway, in which people with low socioeconomic level (OR = 1.79; 95%CI: 1.30; 2.46) and education (OR = 1.70; 95%CI: 1.27; 2.27) have a higher odds of belonging to the “metabolic” pattern. On the other hand, these individuals presented lower odds (OR = 0.48; 95%CI: 0.33; 0.69 and OR = 0.40; 95%CI: 0.28; 0.56, respectively) to belonging to the “allergic” pattern.

**Table 7:** Association between the patterns of morbidities and demographic and socioeconomic factors in individuals at 40 years of age. 1982 Pelotas (Brazil) birth cohort, 2022/2023.

Relatively healthy (reference)	Endocrine/Cardiovascular		Allergic/Respiratory	
	Crude OR (95%CI)	Adjusted OR (95%CI)	Crude OR (95%CI)	Adjusted OR (95%CI)
Sex				
Male	1.00 Reference	1.00 Reference	1.00 Reference	1.00 Reference
Female	1.08 (0.88; 1.33)	1.06 (0.86; 1.30)	1.09 (0.90; 1.32)	1.07 (0.88; 1.29)
Skin color				
White	1.00 Reference	1.00 Reference	1.00 Reference	1.00 Reference
Black	1.46 (1.12; 1.89)	1.46 (1.12; 1.90)	0.62 (0.46; 0.84)	0.62 (0.46; 0.85)
Mixed-race	1.21 (0.87; 1.68)	1.21 (0.87; 1.68)	0.98 (0.72; 1.33)	0.98 (0.72; 1.34)
Socioeconomic level (ABEP)				
AB	1.00 Reference	1.00 Reference	1.00 Reference	1.00 Reference
C	1.41 (1.12; 1.78)	1.42 (1.12; 1.79)	0.76 (0.62; 0.92)	0.77 (0.62; 0.94)
DE	1.77 (1.29; 2.41)	1.79 (1.30; 2.46)	0.46 (0.32; 0.66)	0.48 (0.33; 0.69)
Education (years of study)				
≥ 12	1.00 Reference	1.00 Reference	1.00 Reference	1.00 Reference
9-11	1.61 (1.27; 2.03)	1.61 (1.27; 2.04)	0.76 (0.61; 0.93)	0.76 (0.62; 0.95)
0-8	1.70 (1.29; 2.26)	1.70 (1.27; 2.27)	0.39 (0.27; 0.55)	0.40 (0.28; 0.56)

95%CI: 95% confidence interval; ABEP: Brazilian Association of Research Companies; OR: odds ratio.

Note: adjusted for sex and skin color.

Table 1 shows the absolute standardized differences between losses and non-losses before and after inverse probability weighting analysis. After weighting from the propensity score to be a loss, we could observe that the standardized differences were close to zero. Table 2 shows the results of the association between multimorbidity and demographic and socioeconomic factors after the inverse probability weighting. Comparing the results from Table 5, it is possible to verify that the directions and magnitudes of the associations were quite similar and the results may not be affected by possible selection bias.

## Discussion

This study showed that most participants had at least one morbidity. Black individuals were more likely to be in a higher category of number of cardiovascular morbidities and less likely to have allergic/respiratory morbidities. Socioeconomic status was positively associated with the odds of being in a higher category of number of allergic/respiratory morbidities. Notably, those with lower socioeconomic conditions had higher odds of having more cardiovascular and musculoskeletal morbidities. Endocrine morbidities were not associated with demographic and socioeconomic variables. Three patterns of morbidities were identified: relatively healthy, endocrine/cardiovascular and allergic/respiratory. The directions of the association between demographic and socioeconomic factors and the patterns of morbidities were similar to the associations with the number of morbidities.

Socioeconomic status is strongly associated with the development of NCDs via different mechanisms occurring depending on the type of morbidity ^8^ ^,^ ^9^. Overall, poorer individuals have more morbidities mainly because of the reduced likelihood of accessing health care services, contributing to an increase in the number of morbidities that these individuals may have ^15^.

Regarding cardiovascular diseases, poor eating habits, sedentary behavior, binge drinking and tobacco use are key modifiable behavioral risk factors that are strongly related with socioeconomic status and income ^1^ ^,^ ^16^. A study based on data from the PNS showed that adults with low socioeconomic status had higher burden of cardiovascular disease ^17^. Likewise, using data from a national population-based study with Danish adults, Larsen et al. ^18^ reported that low educational attainment was associated with an increased likelihood of belonging to a cardiovascular multimorbidity pattern.

Regarding musculoskeletal morbidities, the association with low socioeconomic status may be related to these individuals being more likely to be exposed to unhealthy and hazardous work environments ^19^. Conditions like arthritis, arthrosis and chronic back pain are more prevalent in this context due to maintaining uncomfortable postures for prolonged periods; vibrations caused by the use of certain machinery; performing repetitive movements; lifting excessive loads, and working excessively long shifts ^20^. A study carried out in Iran also reported an inversely proportional association between education level and the number of musculoskeletal morbidities ^21^. Similarly a study carried out with individuals older than 60 years from southern Brazil reported that socioeconomic and educational level were inversely associated with musculoskeletal morbidities ^22^.

Conversely to the observed for cardiovascular and musculoskeletal morbidities, the odds of being in a higher category of number of allergic/respiratory morbidities were higher among subjects with high socioeconomic condition. Similar findings have been reported in studies carried out in other countries ^7^ ^,^ ^23^. Conversely, a study in the United States found that adults from low socioeconomic status had higher respiratory morbidities ^24^. Studies with older adults reported similar findings, showing that lower socioeconomic status was associated with higher respiratory morbidities ^25^ ^,^ ^26^. The hygiene hypothesis suggests that excessively clean environments, which are more frequent at higher socioeconomic levels, may be associated with higher prevalences of allergies and respiratory morbidities. This association is attributed to diminished exposure to microorganisms, which may impair the proper development of the immune system ^27^.

A multi-country study - including China, India, Poland, South Africa and Spain - found multimorbidity patterns similar to our study; respiratory and metabolic patterns were identified ^28^. The study found similar patterns in low-middle and high-income countries, suggesting that factors such as unplanned urbanization, aging trends, unhealthy eating habits, sedentary lifestyle, and globalization of product marketing may influence the increase of multimorbidity ^28^. In Brazil, respiratory and cardiovascular patterns were identified also showing that higher education and income were associated with reduced risk of having multiple morbidities ^29^.

Beyond socioeconomic factors, our results indicate racial disparities in specific groups of morbidities. Although previous studies, such as ELSA-Brasil, have reported racial inequalities in the overall prevalence of multimorbidity, our findings suggest that the disparities may differ depending on the morbidity group ^10^. In our study, participants who self-identified as black had a higher risk of cardiovascular diseases and a lower risk of allergic and respiratory conditions compared to those who identified as white. Previous studies have found that the greater burden of cardiovascular diseases among black participants may be related to cumulative exposure to adverse social determinants of health, including socioeconomic disadvantage and limited access to preventive care ^30^ ^,^ ^31^ ^,^ ^32^.

Although evidence suggests sex-based differences in the occurrence of chronic conditions, particularly at older ages ^28^ ^,^ ^33^ ^,^ ^34^ ^,^ ^35^, no statistically significant associations were found between sex and the presence of multiple morbidities in our sample. Previous studies with adults aged 20 and older have found that sex-based differences in morbidity occurrence were minimal before approximately age 60, but became more pronounced with advancing age ^33^ ^,^ ^35^. Possibly, such differences are still slight in the age range evaluated in our study.

Our results also showed a high burden of morbidities among participants at 40 years. Although multimorbidity is more frequently studied in older populations, national studies indicate that around 30% of Brazilian adults have two or more chronic conditions, with prevalence increasing progressively with age ^36^ ^,^ ^37^. The *Brazilian Longitudinal Study of Aging* (ELSI-Brazil, acronym in Portuguese) also reported a high prevalence of multimorbidity (over 58%) even among relatively younger individuals aged 50 to 59 years ^38^. The high prevalence highlighted by many studies could be associated with stressful life events. Personal- and family-related stress increases the likelihood of the onset of chronic diseases, in addition to contributing to the worst prognosis of multiple morbidities. Furthermore, the accumulation of these stressful events throughout life may have even greater consequences ^39^ ^,^ ^40^. The high frequency of morbidities at age 40 observed in our study suggests the early onset of chronic conditions, particularly among more vulnerable individuals, as shown in our analyses.

This study has some limitations. Firstly, the morbidity data are based on self-reported medical diagnoses, which may result in underreporting, especially among participants with lower access to health care services. Second, the data at age 40 come from a birth cohort, and losses to follow-up are common, making the study susceptible to selection bias if the losses are differential. However, after applying the inverse probability weighting, we could verify that there were no differences between losses and non-losses, suggesting that selection bias probably did not affect our estimates (Tables 1 and 2). Finally, some aspects of the sample may limit the generalizability of the findings, which should be considered when interpreting the results. The distribution of ABEP socioeconomic classes in our sample differs from that of the general Brazilian population, possibly underrepresenting more disadvantaged groups. Additionally, participants who identified as yellow and Indigenous were excluded due to small sample sizes, which could reduce the statistical power for detecting differences in these subgroups.

The association of low socioeconomic status with higher morbidity reinforces the need for targeted interventions to reduce social inequalities. Few studies evaluated these health outcomes in adulthood. Given the high prevalence of morbidity observed from a young age, regardless of socioeconomic level, it is important to investigate this age group further.

## Data Availability

The research data are avaliable upon request to the corresponding author.
